# Systematic Analysis of the Maize PHD-Finger Gene Family Reveals a Subfamily Involved in Abiotic Stress Response

**DOI:** 10.3390/ijms161023517

**Published:** 2015-09-30

**Authors:** Qianqian Wang, Jinyang Liu, Yu Wang, Yang Zhao, Haiyang Jiang, Beijiu Cheng

**Affiliations:** Key Laboratory of Crop Biology of Anhui Province, Anhui Agricultural University, No. 130, Changjiang West Road, Hefei 230036, China; E-Mails: qqwangahau@163.com (Q.W.); liujinyang315@163.com (J.L.); yuwangahau@163.com (Y.W.); zhaoyang2013@ahau.edu.cn (Y.Z.)

**Keywords:** PHD family, genome-wide analysis, duplication, maize, abiotic stress

## Abstract

Plant homeodomain (PHD)-finger proteins were found universally in eukaryotes and known as key players in regulating transcription and chromatin structure. Many PHD-finger proteins have been well studied on structure and function in animals. Whereas, only a few of plant PHD-finger factors had been characterized, and majority of PHD-finger proteins were functionally unclear. In this study, a complete comprehensive analysis of maize PHD family is presented. Sixty-seven PHD-finger genes in maize were identified and further divided into ten groups according to phylogenetic analysis that was supported by motif and intron/exon analysis. These genes were unevenly distributed on ten chromosomes and contained 12 segmental duplication events, suggesting that segmental duplications were the major contributors in expansion of the maize PHD family. The paralogous genes mainly experienced purifying selection with restrictive functional divergence after the duplication events on the basis of the *K*a/*K*s ratio. Gene digital expression analysis showed that the PHD family had a wide expression profile in maize development. In addition, 15 potential stress response genes were detected by promoter *cis*-element and expression analysis. Two proteins ZmPHD14 and ZmPHD19 were located in the nucleus. These results provided a solid base for future functional genome study of the PHD-finger family in maize and afforded important clues for characterizing and cloning potentially important candidates in response to abiotic stresses.

## 1. Introduction

The zinc-finger protein is a kind of protein with a “finger” domain, and the zinc-finger domains are rich in cysteine or histidine. Conservative cysteine residues and histidine residues can stabilize the normal space structure by combining zinc ions. According to the arrangement characteristics of conservative cysteine residues and histidine residues, Zinc-finger proteins are divided into several types including Really Interesting New Genes (RING), LIM (Lin11, Isl-1 and Mec-3), plant homeodomain (PHD), *etc.* [1]. The plant homeodomain (PHD) fingers are widely distributed and conserved throughout eukaryotes and most proteins are located in the nucleus, which has been found from yeast to man [2]. The PHD proteins contain one or more PHD-finger domains those are composed of about 60 amino acids with the structural characteristic of Cys4-His-Cys3, which is similar to RING and LIM domains [2,3]. The number of bases between the cysteine and histidine are relatively conserved, as is that between the cysteine residues, and the second amino acid residue before the last pair of cysteines is typically an aromatic amino acid such as tryptophan [4]. These core amino acids residues combine with two zinc ions and are separated by three loop regions [5]. Recent studies show that the PHD-finger domain can specifically bind to histone H3 trimethylated at lysine [6].

Since the first PHD-finger protein HAT3.1 was found in *Arabidopsis* [5], more and more PHD-finger proteins were identified as involved in a number of physiological and biochemical process in fungus, animals or plants, including the regulation of chromatin structure and transcription, *etc.* [7]. Through combination with nucleosomal histones, some PHD-finger proteins regulate the chromatin state [8]. For example, in *Arabidopsis*, the PHD-finger protein DUET was required for organization and progression of chromosomes during male meiosis [9]; VIM1, an *Arabidopsis* PHD-finger protein could associate with histone to participate in the regulation of the chromatin state [10]; MMD1, encoding a PHD domain-containing protein, is expressed preferentially in male meiocytes, which are involved in chromatin remodeling and transcriptional events required for successful progression during meiosis [11]. The latest research shows that PHD proteins play important roles in methylation maintenance [12,13]. The mammals PHD protein UHRF1 is crucial in the maintenance of DNA methylation [14] and ATX1 and ATX2 with the activity of histone methyltransferase regulated the transcription of certain genes [15]. The PHD-finger protein is reported to mediate the protein–protein interactions [16], which are already known to be involved in various human diseases [17], for example, UHRF1, a PHD protein plays an important role in breast carcinogenesis by forming a complexwith other proteins [18]. In addition, some PHD-fingers were considered to take part in the regulation of ubiquitination [19], and were detected as binding targets of rare phosphatidylinositol phosphates (*PtdInsPs*) which play crucial roles in DNA damage signaling [20]. Furthermore, a number of PHD proteins play significant roles in plant response to abiotic stresses; six soybean PHD proteins were identified as regulators of abscisic acid (ABA) signaling and conferred salt tolerance in transgenic *Arabidopsis* [21].

Although the number of PHD-finger homologs identified is increasing in different species, most putative PHD family members remain unrecognized and uncharacterized in each organism, and few comprehensive analyses of the PHD family in evolution have been conducted. It would be of great significance to get a better understanding of the multiple PHD family members, because of their potentially important functions in many central biological processes. Maize is an important cereal crop and has become a model plant for the study of genetics, evolution and other basic biological research [22]. The availability of the maize genome sequences has provided an excellent opportunity for whole-genome annotation, classification and comparative genomics research [23,24]. In this paper, we performed a comprehensive analysis for PHD family in maize genome, including the phylogenetic relationship, gene structure, chromosomal localization and gene duplication analysis. Seventeen maize PHD-finger factors that might participate in the process of various abiotic stresses were identified, and their expression patterns were detected in response to ABA, PEG and NaCl treatment. All these results provide a better understanding of the maize PHD-finger family on evolutionary history and functional mechanisms.

## 2. Results

### 2.1. Identification of PHD Proteins in Maize

To identify the *PHD* genes in the maize genome, the consensus protein sequences of the PHD-finger Hidden Markov Model (HMM) profile were employed as a query to search against the maize genome database with the BLASTP program. A total of 95 candidate PHD-finger protein sequences were identified in maize. Based on sequences similarity analysis, 28 redundant PHD-finger sequences were discarded. In order to confirm the reliability of putative PHD-finger members in maize, the amino acid sequences of the 67 proteins were searched for the presence of PHD domains with Pfam and SMART programs. The results showed that all of the 67 non-redundant PHD-finger proteins in maize contained the PHD conserved domain. Subsequently, the 67 PHD genes were named *ZmPHD1* to *ZmPHD67* according to their locations in chromosomes (Table 1). The length of all maize PHD proteins was between 72 and 2379 amino acids with an average of 756 amino acids. The detailed information of *ZmPHD* genes is listed in Table 1, including chromosome location, protein length (aa) and gene identifier.

**Table 1:** The detailed information of maize PHD family members.

Gene Name	Gene Identifier	Protein Size (aa)	5′ End	3′ End	Chromosme
*ZmPHD1*	GRMZM5G862565	1579	100,335,533	100,348,389	I
*ZmPHD2*	GRMZM5G866423	287	209,536,012	209,538,518	I
*ZmPHD3*	GRMZM5G813111	252	276,110,228	276,115,825	I
*ZmPHD4*	AC225147.4	250	292,875,307	292,879,433	I
*ZmPHD5*	GRMZM2G403562	1900	144,629,199	144,637,545	II
*ZmPHD6*	GRMZM2G013936	751	155,881,115	15,590,458	II
*ZmPHD7*	GRMZM2G047316	241	158,280,766	158,284,014	II
*ZmPHD8*	GRMZM2G409224	1566	198,098,543	198,115,319	II
*ZmPHD9*	GRMZM2G181158	345	201,127,832	201,131,690	II
*ZmPHD10*	GRMZM2G158194	556	9,042,243	9,044,511	III
*ZmPHD11*	GRMZM2G085266	971	33,200,782	32,090,831	III
*ZmPHD12*	GRMZM2G069886	1214	114,434,585	114,444,762	III
*ZmPHD13*	GRMZM2G081350	818	133,742,782	133,749,539	III
*ZmPHD14*	GRMZM2G153087	257	135,907,092	135,912,402	III
*ZmPHD15*	GRMZM2G080917	257	170,106,279	170,110,892	III
*ZmPHD16*	GRMZM2G025703	173	171,031,966	171,035,433	III
*ZmPHD17*	GRMZM2G314546	1577	197,392,618	197,405,889	III
*ZmPHD18*	GRMZM2G068331	697	10,228,455	10,233,101	IV
*ZmPHD19*	GRMZM2G008259	172	11,389,157	11,413,875	IV
*ZmPHD20*	GRMZM2G059266	558	40,354,679	40,358,758	IV
*ZmPHD21*	GRMZM5G871463	72	79,000,726	79,005,980	IV
*ZmPHD22*	GRMZM2G107807	255	120,625,503	120,629,020	IV
*ZmPHD23*	GRMZM2G067019	418	136,070,311	136,101,261	IV
*ZmPHD24*	GRMZM2G385338	1812	234,187,863	234,200,009	IV
*ZmPHD25*	GRMZM2G391413	1322	239,318,755	239,331,611	IV
*ZmPHD26*	GRMZM2G110952	1166	2,576,103	2,593,650	V
*ZmPHD27*	GRMZM2G087482	1376	7,443,884	7,459,423	V
*ZmPHD28*	GRMZM2G466292	527	65,425,225	65,434,544	V
*ZmPHD29*	GRMZM2G466270	1290	65,442,955	65,456,381	V
*ZmPHD30*	GRMZM2G063864	255	174,409,725	174,413,665	V
*ZmPHD31*	GRMZM2G045544	727	203,646,189	203,650,857	V
*ZmPHD32*	GRMZM2G368206	1465	210,502,142	210,518,679	V
*ZmPHD33*	GRMZM2G039895	555	1,537,396	1,541,397	VI
*ZmPHD34*	GRMZM2G134214	849	54,775,354	54,785,293	VI
*ZmPHD35*	GRMZM2G103230	1147	58,263,970	58,269,763	VI
*ZmPHD36*	GRMZM2G473258	321	94,516,049	94,518,519	VI
*ZmPHD37*	GRMZM2G168249	808	107,094,897	107,103,424	VI
*ZmPHD38*	GRMZM2G330024	1108	109,391,739	109,405,354	VI
*ZmPHD39*	GRMZM2G148810	253	124,705,685	124,710,238	VI
*ZmPHD40*	GRMZM2G016817	256	147,643,515	147,650,880	VI
*ZmPHD41*	GRMZM2G412492	999	8,826,124	8,831,882	VII
*ZmPHD42*	GRMZM2G097726	219	13,348,616	13,356,443	VII
*ZmPHD43*	GRMZM2G013794	976	72,242,570	72,287,055	VII
*ZmPHD44*	GRMZM2G091265	212	105,907,436	105,911,988	VII
*ZmPHD45*	GRMZM2G316191	2379	150,159,272	150,178,742	VII
*ZmPHD46*	GRMZM2G128176	1712	171,564,760	171,575,198	VII
*ZmPHD47*	GRMZM2G434715	771	174,765,898	174,774,731	VII
*ZmPHD48*	GRMZM5G893976	241	15,274,043	15,278,560	VIII
*ZmPHD49*	GRMZM2G372928	759	25,354,740	25,391,185	VIII
*ZmPHD50*	GRMZM2G158918	256	103,454,290	103,460,620	VIII
*ZmPHD51*	GRMZM2G017142	253	125,536,101	125,539,093	VIII
*ZmPHD52*	GRMZM2G335720	836	137,142,057	137,164,518	VIII
*ZmPHD53*	GRMZM2G170412	870	147,718,464	147,724,348	VIII
*ZmPHD54*	GRMZM2G149587	381	156,906,971	156,912,759	VIII
*ZmPHD55*	GRMZM2G172001	255	164,744,187	164,748,517	VIII
*ZmPHD56*	GRMZM2G455243	852	12,345,652	12,367,654	IX
*ZmPHD57*	GRMZM2G156129	108	29,157,055	29,158,213	IX
*ZmPHD58*	GRMZM2G472428	1990	30,050,060	30,074,947	IX
*ZmPHD59*	GRMZM5G889372	868	136,475,636	136,480,652	IX
*ZmPHD60*	GRMZM2G178072	249	152,186,947	152,194,716	IX
*ZmPHD61*	GRMZM2G314661	1869	4,871,817	4,880,152	X
*ZmPHD62*	GRMZM2G365888	819	27,266,656	27,273,862	X
*ZmPHD63*	GRMZM2G115424	245	31,307,377	31,312,172	X
*ZmPHD64*	GRMZM2G404426	421	65,737,118	65,752,360	X
*ZmPHD65*	GRMZM2G156088	249	119,469,068	119,473,971	X
*ZmPHD66*	GRMZM2G050495	248	119,702,189	119,707,621	X
*ZmPHD67*	GRMZM2G038050	1339	149,547,787	149,558,325	X

### 2.2. Phylogenetic and Structural Analysis

**Figure 1:**
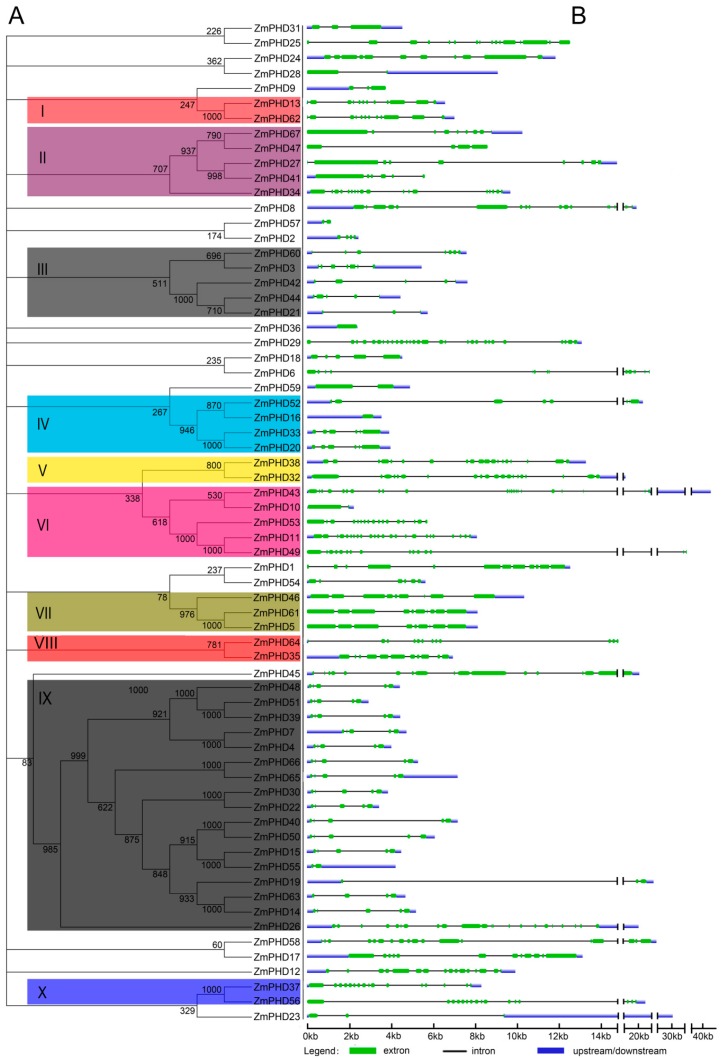
Phylogenetic relationships and gene structure of maize *PHD* genes. (**A**) An unrooted tree is generated with the MEGA4.0 software using the full-length amino acid sequences of the 67 maize PHD proteins by the Neighbor-Joining (NJ) method, with 1000 bootstrap replicates. The tree shows ten major phylogenetic groups (group I to X) indicated with different colored backgrounds; and (**B**) Exon/intron organization of maize *PHD* genes. Green boxes indicate exons and grey lines represent introns, and the untranslated regions (UTRs) are indicated by blue boxes. The sizes of exons and introns can be estimated using the scale at the bottom.

To gain further insights into the evolutionary relationships of maize *PHD* genes, we constructed an unrooted phylogenetic tree with 67 maize PHD-finger proteins sequences (Figure 1A), and investigated the exon/intron structures of individual *ZmPHD* genes by comparing their cDNA sequences with corresponding genomic DNA sequences (Figure 1B). To facilitate the research and analysis, the ZmPHD family was divided into 10 groups (group I to group X) according to the bootstrap values (>500) in the Neighbor-Joining (NJ) tree, while some ZmPHD members were beyond the 10 groups because of low bootstrap values (<500) of the NJ tree, which was also shown in previous study [24]. Group IX contained 17 members and was the largest clade of all the groups, which represented the 25.37% of the total ZmPHD proteins. Whereas, groups I, V, VIII and X only had two members.

Observation of the exon/intron structure in maize *PHD* genes (Figure 1B) revealed the number of introns varied from 1 to 28, with the exception of *ZmPHD16* and *ZmPHD36* having no introns, and *ZmPHD29* having the most introns (28) among the *ZmPHD* family. In addition, most maize *PHD* genes clustered in the same group shared highly similar exon/intron distribution patterns, including the exon length and intron numbers. For example, two genes locating in group X both had 15 introns, and the members belonging to group VII contained eight introns. Likewise, most genes in group IX possessed four introns except *ZmPHD19*, *-26*, *-55*, *-63*, and *-65*, which had 2, 19, 1, 3 and 5 introns, respectively. By comparison, the genes in groups II and III showed great diversity in exon length as well as the intron numbers (Figure 1B). In summary, the exon/intron structures of maize *PHD* genes were basically in line with the phylogenetic relationship.

### 2.3. The Sequence Analysis of Maize PHD-Finger Domain and Motifs

**Figure 2:**
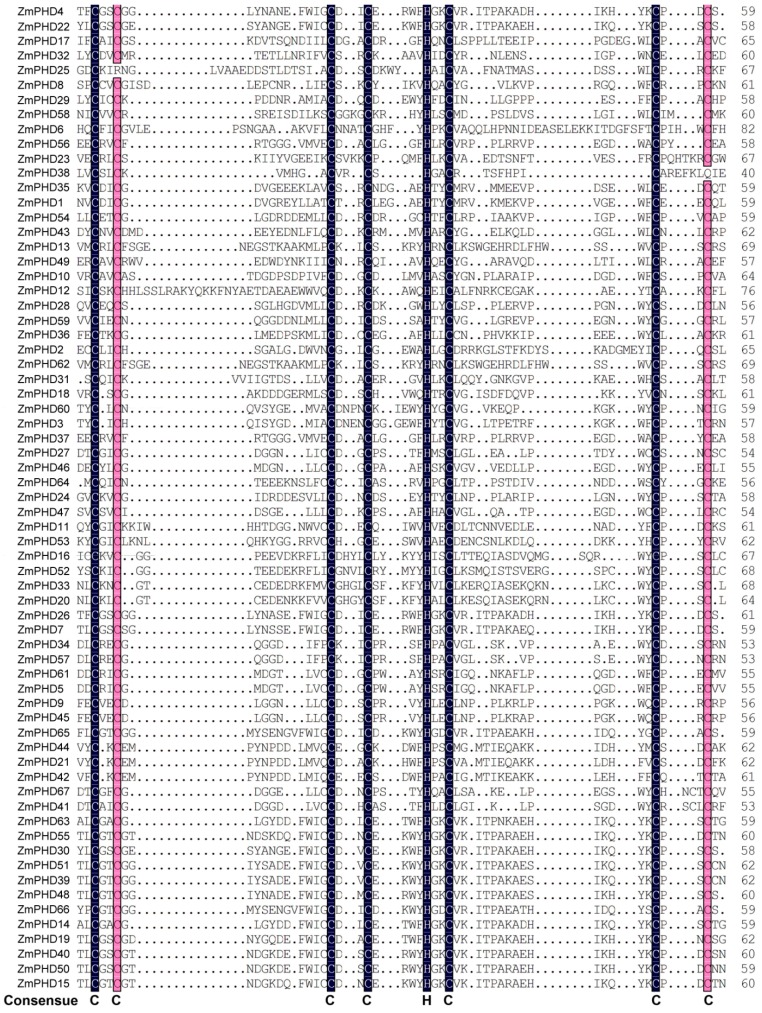
Multiple sequence alignment of PHD-finger domain of 67 maize PHD proteins. The shading of the alignment presents identical residues in black and similar residues in red, and the high conserved amino are marked at the bottom. The PHD-finger domain of each ZmPHD members is corresponding to the first PHD motif in the upstream of each PHD protein in Figure 3, respectively.

To have a further understanding of the similarity between the maize PHD-finger domains, we aligned 67 maize PHD-finger domain sequences (Figure 2). The length of the 67 sequences varied from 40 to 60 amino acids, and the highlighted figures in black and red areas were the Zn ion binding sites with seven cysteines and one histidine residue. The PHD-finger domain was conservative because of the Zn ion binding sites contributing to the structural stability of domain. Based on the result of sequence alignment, we found the maize PHD-finger domain consensus sequence was C–X(1–2)-C–X (8–19)-C–X(2–4)-C–X(4–6)-H-X2-C–X(11–26)-C–X(2–3)-C, which was basically consistent with the previous findings [25].

**Figure 3:**
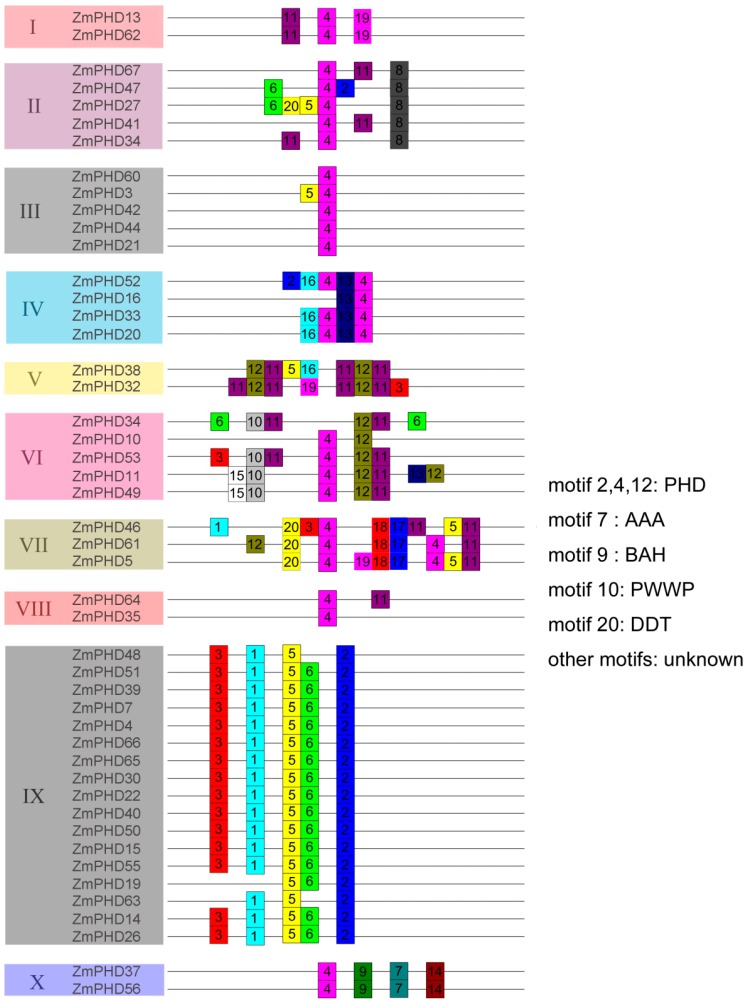
Distribution of conserved motifs in maize PHD members. All motifs were identified by MEME using the complete amino acid sequences of ZmPHD proteins. Different motifs are indicated by different colors boxes numbered 1–20, and the length of each box in the proteins does not represent the actual motif size. The annotation of each motif is listed on the right. PHD: plant homeodomain; AAA: ATPases Associated with diverse cellular Activities; BAH: Bromo-adjacent Homology domain; PWWP: Pro-Trp-Trp-Pro motif; DDT: DNA binding homeobox and Different Transcription factors.

To gain more insight into the diversity of motif compositions among ZmPHDs, maize PHD proteins from group I to X were subjected to the MEME tool. Twenty conserved motifs that designated as motif 1 to motif 20 in ZmPHD proteins were identified (Figure 3). The details of each motif are shown in Table S1. In addition, the SMART and Pfam were employed to annotate the 20 identified putative motifs (Figure 3). Furthermore, to make the MEME results concise and clear, two or more adjacent motifs, representing the same domain, were merged and displayed as one domain district. Of the 20 motifs, half remained as function unknown in SMART and Pfam databases. Motifs 2, 4 and 12 stood for the conserved PHD domain and were found one or more times among all the ten group members. Most maize PHD proteins within the same groups shared highly similar motif compositions and distribution, which implied the ZmPHD members within the same groups might share similar functions. However, great difference waere also observed between different groups. For example, proteins in group I possessed motifs 4, 9 and 11, while group IX members contained motifs 1, 2, 3, 5 and 6 (Figure 3). Besides, some motifs that were exclusively observed in a particular group, suggested that these motifs might contribute to specific functions of that group. For instance, motif 9 represents the Bromo-adjacent Homology domain (BAH) domain that existed only in group X members, and shown to play an important role in DNA methylation, replication and transcriptional regulation [26]; motif 10 is a PWWP domain, named after a conserved Pro-Trp-Trp-Pro motif, only found in group VI members except the ZmPHD10, which is methyl-lysine recognition motif that is involved in a series of cellular processes by binding to histone-4 methylated at lysine-20, such as maintaining genome stability and regulating cell-cycle progression [27,28]; moreover, motif 20 representing the DNA binding homeobox and Different Transcription factors (DDT) domain was merely present in the group VII and ZmPHD27 and motif 7 representing the ATPases Associated with diverse cellular Activities (AAA) domain was only belong to group VII as well as motif 9 (Figure 3). The motif distribution among ZmPHD proteins further supported the closely evolutionary relationships among ZmPHD proteins as well as the reliability of the phylogenetic analysis.

### 2.4. Chromosomal Locations and Duplications of ZmPHDs

**Figure 4:**
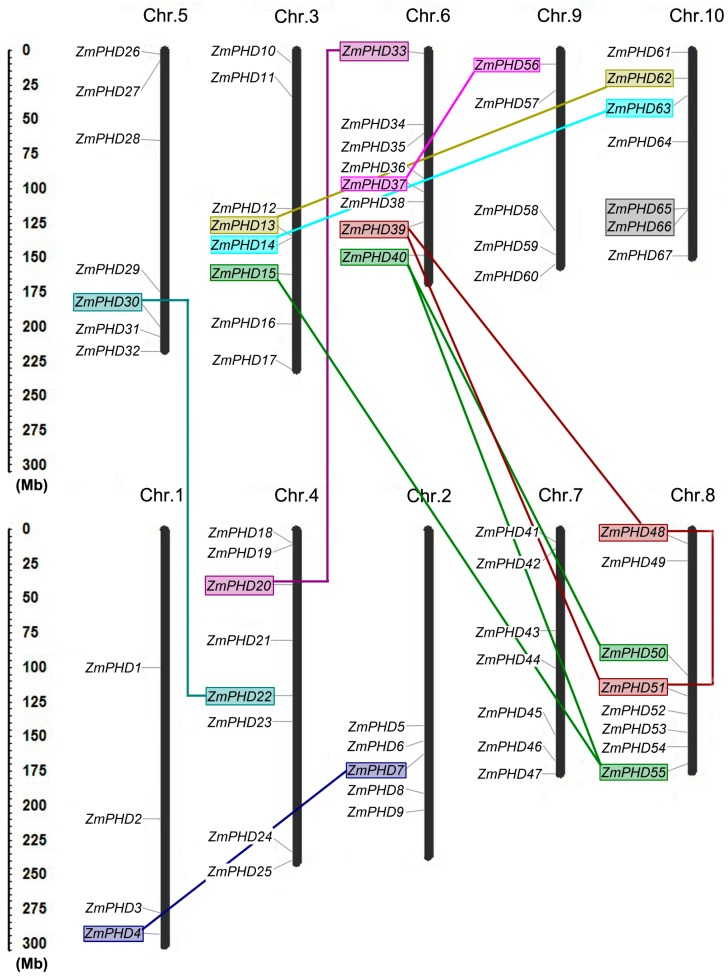
Chromosomal locations of maize *PHD* genes. The left scale represents the megabases (Mb). Chromosome numbers are shown at the top of each vertical black bar. The rough location of each maize *PHD* genes is marked whit the grey line that link the gene names with the chromosome bars. The segmental duplication gene pairs are marked with color boxes and joined by corresponding color lines, and the grey box indicates the tandem duplication gene pairs.

Sixty-seven maize *PHD* genes were placed on 10 maize chromosomes according to their positions in the maize genome (Figure 4), which showed that maize *PHD* genes were unevenly distributed on the 10 maize chromosomes. Chromosome 3, 6 and 8 contained highest number of *ZmPHD* genes (8); only four members were assigned on chromosome 1. The precise location of all *PHD* genes on the ten maize chromosomes is listed in detail in Table 1. Distribution of these *PHD* genes on individual chromosome was also irregular; for example; genes on chromosome 2 were distributed on the lower end of the arm; while genes were basically equally distributed on chromosome 7 (Figure 4).

Gene duplication events, including tandem and segmental duplications, are of great importance to expand the number of gene family members [29]. Potential duplication events were investigated with the purpose of elucidating the expanded mechanism of the maize *PHD* gene family, which had occurred during the process of evolution. Based on the phylogenetic and comparative analysis of the *ZmPHD* genes, 12 gene pairs (*ZmPHD13*/*62*, *ZmPHD20*/*33*, *ZmPHD51*/*39*, *ZmPHD48*/*39*, *ZmPHD48*/*51*, *ZmPHD4*/*7*, *ZmPHD22*/*30*, *ZmPHD40*/*50*, *ZmPHD15/55*, *ZmPHD63*/*14*, *ZmPHD37*/*56*, *ZmPHD40*/*55*) were identified to be involved in the segmental duplication events. Among the 12 segmental duplication pairs, the high frequency of segmental duplication appeared between chromosomes 6 and 8, which contained four segmental duplications. It is noteworthy that only one gene pair *ZmPHD65*/*66* was involved in tandem duplication. These results suggest that segmental duplication events are the main gene duplication events in the maize PHD-finger family, and might play major roles in the amplification of the maize PHD family, which is consistent with previous research [24].

To explore the selection in duplication and divergence of PHDs in maize, the non-synonymous (*K*a), synonymous (*K*s) and *K*a/*K*s were calculated for each pairs of duplicated *ZmPHD* genes. The *K*a and Ks were used to examine the course of divergence after duplication, and the *K*a/*K*s ratio was applied to measure selective pressure of duplicated gene pairs. Generally, the value of *K*a/*K*s > 1 indicated positive selection that accelerated the evolution; the *K*a/*K*s ratio = 1 signified neutral selection; while *K*a/*K*s ratio < 1 stood for negative selection or purifying selection. The value of *K*a/*K*s of 13 duplicated gene pairs in maize varied from 0.084 to 0.764 with an average of 0.286.; in addition, the majority of them were less than 0.3, which indicated that the duplicated *ZmPHD* genes were under strong negative selection during evolution (Table 2). Based on the estimations for *K*s, the time of 13 duplicated evens were calculated with a substitution rate of 6.5 × 10^−9^ substitutions per site per year [30], which were ranged from 7.38 to 74.53 million years (Mya) (Table 2).

**Table 2:** *K*a/*K*s analysis and divergence time estimated for maize duplicated PHD paralogs.

Paralogous Pairs	*K*s	*K*a	*K*a/*K*s	Duplication Date (MY)	Duplicate Type
*ZmPHD4/7*	0.969	0.175	0.181	74.53	segmental
*ZmPHD13/62*	0.148	0.031	0.209	16.07	segmental
*ZmPHD14/63*	0.176	0.026	0.147	13.53	segmental
*ZmPHD15/55*	0.686	0.436	0.635	52.76	segmental
*ZmPHD20/33*	0.23	0.078	0.339	17.69	segmental
*ZmPHD22/30*	0.151	0.019	0.125	11.61	segmental
*ZmPHD37/56*	0.188	0.025	0.132	14.46	segmental
*ZmPHD40/50*	0.131	0.02	0.152	10.07	segmental
*ZmPHD40/55*	0.7	0.535	0.764	53.84	segmental
*ZmPHD48/39*	0.657	0.112	0.17	50.53	segmental
*ZmPHD48/51*	0.627	0.109	0.173	48.23	segmental
*ZmPHD51/39*	0.236	0.02	0.084	18.15	segmental
*ZmPHD65/66*	0.096	0.059	0.614	7.38	tandem

### 2.5. Microsynteny Analysis among Maize, Sorghum and Rice

**Figure 5:**
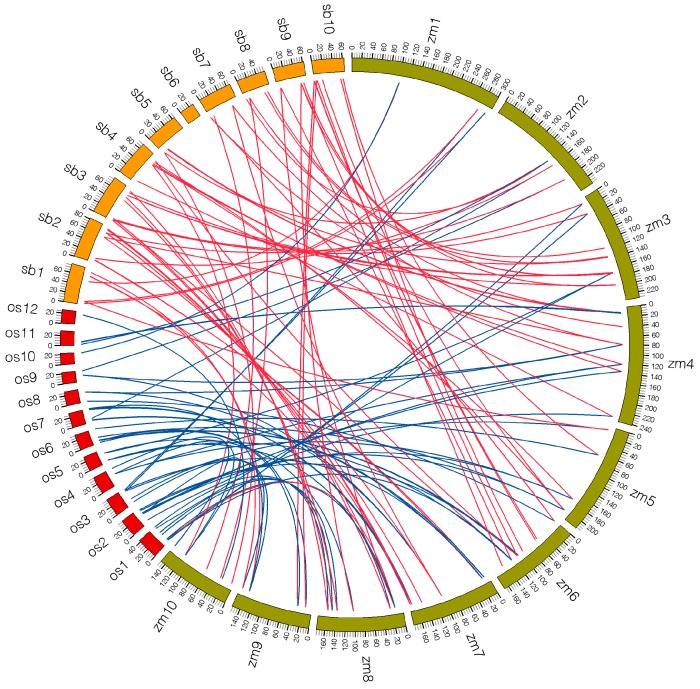
Microsynteny of PHD regions across maize, sorghum and rice. The maize, sorghum and rice chromosomes are shown in different color boxes and labeled zm, sb and os, respectively. Numbers along each chromosome box indicate sequence lengths in megabases. Red lines represent the syntenic relationships between maize and sorghum PHD regions, and blue lines represent the syntenic relationships between maize and rice regions.

To elucidate the evolutionary relationship of PHD families among maize, sorghum and rice, comparative analysis was performed to identify orthologous *PHD* genes. Seventy-one orthologous gene pairs were found between maize and sorghum. Conversely, only 54 orthologous gene pairs were detected between maize and rice (Figure 5, Tables S2 and S3). The number of orthologous genes between maize and sorghum was far greater than that between maize and rice, which might be due to the closer relationship between maize and sorghum [31]. In addition, a portion of collinear gene pairs between maize and sorghum were not identified between maize and rice, such as *ZmPHD35*/*Sb10g001106.1*, *ZmPHD63*/*Sb08g006530.1* and *ZmPHD40*/*Sb09g020610.1* and so on, which implied these orthologous gene pairs appear behind the divergence of the progenitors of maize and rice (Figure 5, Tables S2 and S3). However, two orthologous gene pairs (*ZmPHD57*/*LOC_Os06g01170.1*, *ZmPHD62*/*LOC_Os12g34330.1*) were not found between maize and sorghum that might due to the genes loss in the evolution of sorghum. Furthermore, we also found that two or more PHD genes from sorghum and rice were orthologous of the same maize *PHD* gene; these genes are probably paralogous gene pairs and play important roles in the amplification of *PHD* gene family in the process of evolution. For example, *Sb04g023220.1* and *Sb06g017810.1* are orthologous genes to *ZmPHD30*, as well as *LOC_Os02g48800.1* and *LOC_Os06g20410.1* to *ZmPHD31* (Figure 5, Tables S2 and S3)*.*

### 2.6. Analysis of the Promoters of Potential Abiotic Stress-Responsive PHD Genes

**Figure 6:**
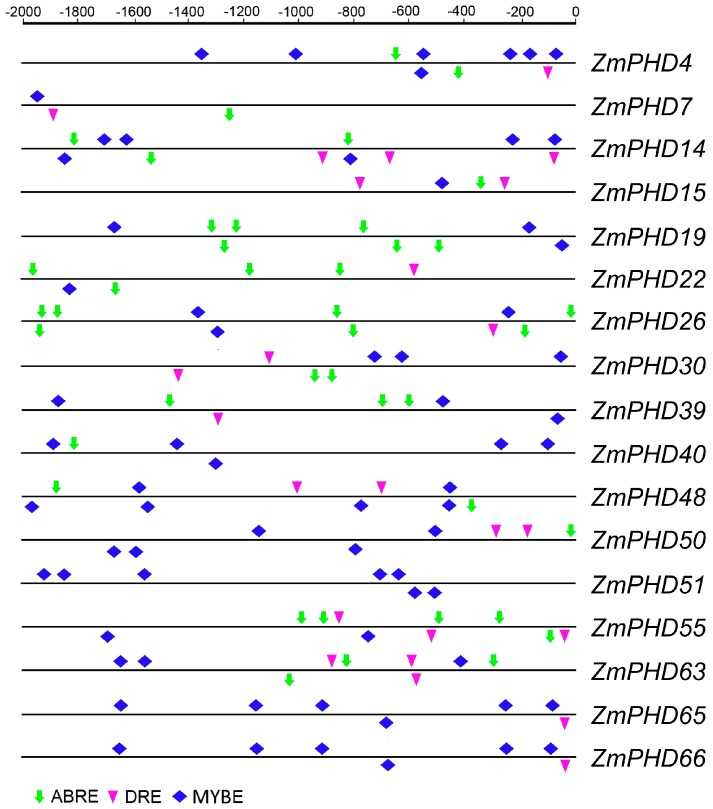
*Cis*-elements in the promoter regions of maize putative stress-responsive *PHD* genes. The stress-responsive *cis*-elements distributed on the sense strand and reverse strand are shown above and below the grey lines, respectively. ABRE, MYB and DRE core sequences are indicated by green thick drop-down arrows, pink triangles and blue squares, respectively. ABRE: ABA responsive element; MYB: MYB-binding site *cis*-element; DRE: dehydration-responsive element.

It was reported that six soybean *GmPHD*-type transcription regulators enhanced stress tolerance in transgenic *Arabidopsis* [21]. We analyzed the conserved motifs for these 6 *GmPHD* genes by MEME, which showed highly similarity with group IX PHD genes (Figure S1). The phylogenetic analysis also showed that they shared closely evolutionary relationship (Figure S1). As proteins functions were closely related to their structures, we conjectured the 17 *ZmPHD* genes might also play a role under adversity stress in maize. This observation prompted us to investigate possible stress-responsive *cis*-elements in the promoter regions of the 17 maize *PHD* genes by searching against the PLACE database. Three types of *cis*-elements, including the ABRE (ABA responsive element), DRE (dehydration-responsive element) and MYB-binding site *cis*-element (MYBE) were detected in current study [32]. The results showed that each of the 17 *PHD* genes contained one or more types of *cis*-elements of ABRE, DRE or in their 2000 bp promoter sequences (Figure 6), which were key elements for stress responsiveness. Although the phylogenetic analysis showed close relationships among these 17 *ZmPHD* genes (Figure S1), we found surprising differences in the numbers of the three *cis*-elements in their promoter regions (Table S4). For example, the promoter regions of *ZmPHD14* and *ZmPHD63* contained multiple putative ABREs, DREs and MYBEs. In contrast, only MYBEs were detected in the promoter of *ZmPHD51*. Moreover, we also found that the *cis*-elements were not conserved in the promoter regions of the five segmental duplicated gene pairs (*ZmPHD22/30*, *ZmPHD40/50*, *ZmPHD14/63*, *ZmPHD40/55*, *ZmPHD48/51*) when compared to the tandem duplicated one (*ZmPHD65/66*). This observation indicated that the five segmental duplication pairs might possess different regulatory feature. In addition, each of these six paralogs contained at least one ABRE or MYBE element, which was the central *cis*-elements signal transduction in response to stress conditions (Figure 6, Table S4). Thus, we concluded that these duplicated genes might share similar regulatory pathway in some respects.

### 2.7. Microarray Expression Profiles of Maize PHD Genes

**Figure 7:**
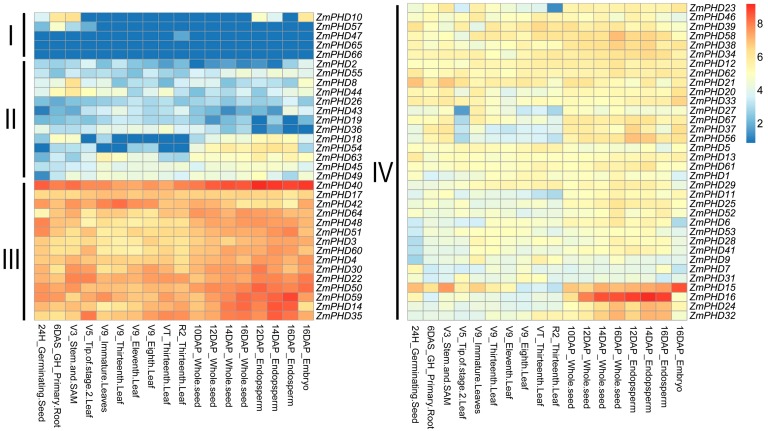
Expression profiles of maize *PHD* genes across different tissues. Genes highly or weakly expressed in the tissues are colored red and blue, respectively. Classes I to IV indicates the different levels of gene *log2*-transformed expression. The gradually change of the color indicates different expression level of *PHD* genes, and yellow color stands for middle expression level.

According to *ZmPHD* genes expression characteristic, 67 transcripts were divided into four parts (Figure 7). Class I to Class IV indicated the levels of gene *log_2_*-transformed expression in different tissues. The heat map shows that most of the 67 *ZmPHD* genes were involved in maize growth and development process, while their expression levels were different. The group IX genes clustering in Classes III and IV appeared to be invariable and highly expression among all tissues except *ZmPHD65* and *ZmPHD66*, which were located in class I with very low or no expression. We found that genes in the same groups with similar expression patterns, such as groups I, III, IV, V, VI, and IX members. However, expression divergence was also obviously observed, for example, a member of group B, *ZmPHD47* was only expressed in R2_Thirteenth Leaf, while the same group gene *ZmPHD27* had a broad expression spectrum, merely without expression in V5_Tip of stage-2 leaf (Figure 7). *ZmPHD16* had considerably high expression level in whole seeds and 12-16DAP-Endosperm, suggesting it probably important role in endosperm growth and development. *ZmPHD40* held high expression at each stage, which implied its crucial functions over the maize life cycle.

### 2.8. Expression Levels of Group IX PHD Genes in Response to Salt, PEG, and ABA Stress Condition

**Figure 8:**
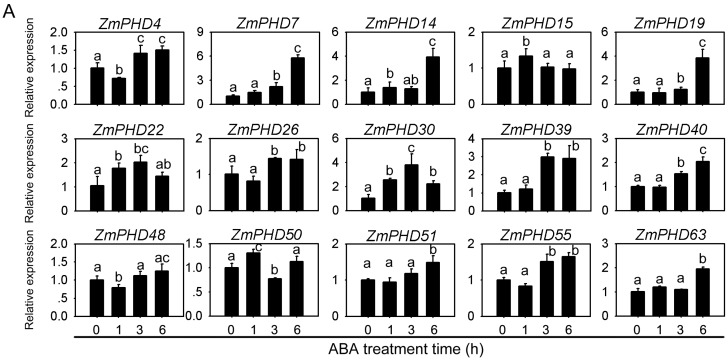

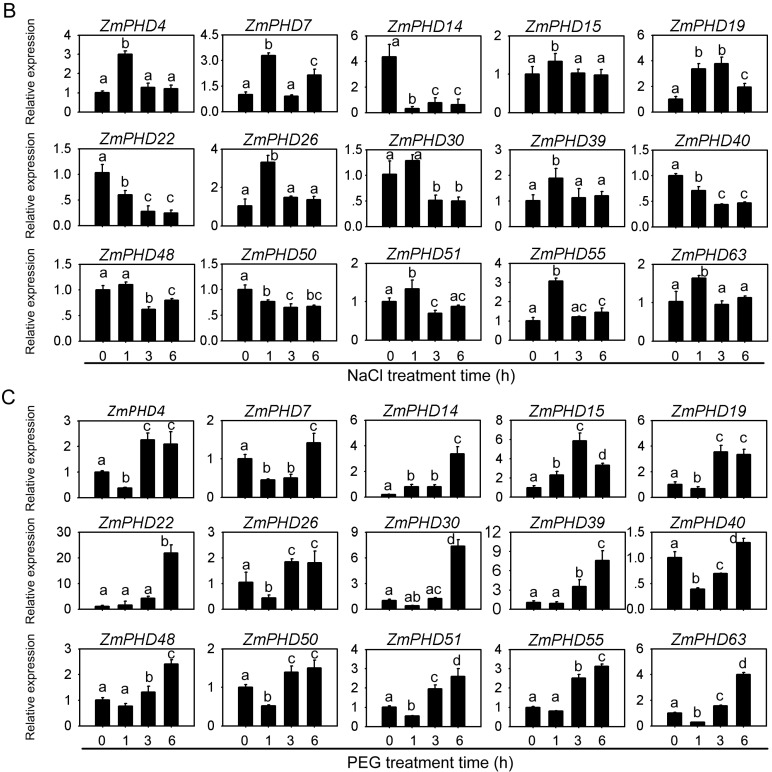
Expression patterns of *PHD* group IX genes in response to drought, NaCl and ABA treatments. The relative expression level of 15 *PHD* group IX genes was examined by the qRT-PCR and normalized with the reference gene *ZmGAPDH*. (**A**) Relative expression of 15 *PHD* genes under ABA treatment at 0, 1, 3, 6 h; (**B**) Relative expression of 15 *PHD* genes under NaCl treatment at 0, 1, 3, 6 h; and (**C**) Relative expression of 15 *PHD* genes under PEG6000 treatment at 0, 1, 3, 6 h. The error bars represent standard deviations (SD), *y*-axes are scales of relative expression level and *x*-axes are the time course of treatments for each condition. Different lowercase letters indicate significant differences at *p <* 0.05 (Duncan’s test).

The analyses of sequences and evolutionary relationships indicated that the 17 *PHD-finger* genes from group IX might participate in response to multiple abiotic stresses including drought and soil salinity (Figure 6 and Figure S1). In this study, Quantitative real-time RT-PCR (qRT-PCR) was employed to investigate the expression patterns of the group IX genes using three-week-old maize leaves under ABA, NaCl, and PEG treatment, respectively. The expression levels of 17 *PHD* genes are shown in Figure 8 except for *ZmPHD65*, *-66*, which were not expressed during the whole treatment stage under three stress treatments (Figure 8). Of the 15 PHD-finger genes, 14 genes were obviously up-regulated in response to ABA stress, besides, *ZmPHD15*, which was up-regulated at 3 h after ABA treatment and down-regulated thereafter. Notably, five out of the 14 up-regulated genes showed great amplitude of variation of expression level (>3-fold), including *ZmPHD7*, *-14*, *-19*, *-30* and *-39*, while the remaining nine genes *ZmPHD4*, *-22*, *-26*, *40*, *-48*, *-50*, *-51*, *-55* and *-63* showed slight expression changes (<2-fold) (Figure 8A). Under NaCl treatment, the expression levels of *ZmPHD15*, *-30*, *-39*, *-48*, *-50*, *-51* and *-63* were mildly regulated (<2-fold), but the other seven genes were highly induced (Figure 8B). Among the 15 PHD-finger genes *ZmPHD14* and *-22* were dramatically down-regulated across all time points. The *ZmPHD4*, *-7*, *-19*, *-26*, and *-55* were strongly up-regulated at the early stage after NaCl treatment, whereas, their expression revealed relatively decreases thereafter, but still higher than the control. All the group IX genes under PEG treatment, especially *ZmPHD22*, *-30*, and *-39* were strongly up-regulated by more than 8- to 30-fold compared to the control (Figure 8C). It is interesting that a large proportion of the up-regulated genes were down-regulated at 1h after PEG treatment, and then reached a peak expression level at 6 h, while the *ZmPHD4*, *-15*, *-19* and *-26* reached the peak expression level at 3 h.

By comparing with the relative expression of *PHD*-finger genes in group IX under ABA, NaCl and PEG treatment, we found *ZmPHD14*, *-19* showed strong induction or repression under all three stress treatments, while the rest genes exhibited highly regulated just in one or two treatment, for *ZmPHD15*, *-48*, *-51*, and *-63* showed highly regulated by PEG stress, but exhibited slightly expression change under other two treatments, *ZmPHD22*, and *-55* were strongly regulated just in PEG and NaCl treatment (Figure 8). Of nine pairs of segmental duplicated genes in the group IX, most exhibited similar expression profiles under three stresses treatment, such as *ZmPHD14/63*, *ZmPHD40/50* following PEG treatment, *ZmPHD39/51* following NaCl treatment and *ZmPHD22/30*, *ZmPHD14/63* following ABA treatment. However, the expression profiling of some segmental duplicated genes was different, for *ZmPHD15/55* under PEG treatment and *ZmPHD4/7* under ABA treatment (Figure 8).

### 2.9. Subcellular Localization of ZmPHD14 and ZmPHD19

**Figure 9:**
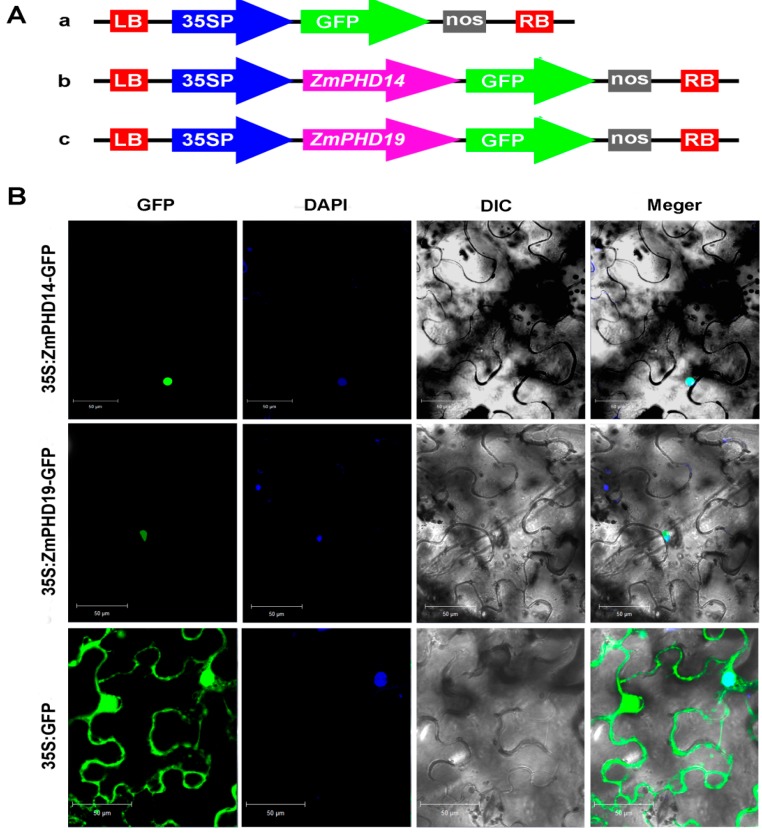
Subcellular localization of ZmPHD14-GFP and ZmPHD19-GFP fusion protein. (**A**) Schematic representation of the 35S:GFP, 35S:ZmPHD14-GFP and 35S:ZmPHD19-GFP fusion constructs used for transient expression; and (**B**) Fusion proteins were transiently expressed under control of the CaMV35S promoter in tobacco leaves and observed under a laser scanning confocal microscope. Green color is GFP protein signal, and blue color represents DAPI stained for nucleus. Bars = 50 μm.

It is reported that a large proportion of PHD-finger proteins are located in nucleus, while some of them are in membrane [33,34]. Six GmPHD proteins (GmPHD1–GmPHD6) were targeted to the nucleus and the PHD domains were required for their nuclear location [21]. Group IX members had a close relationship and similar structures with these GmPHD proteins, hence they might also locate in the nucleus. *ZmPHD14* and *ZmPHD19* showed great expression level changes under NaCl, ABA and PEG treatments, and implied they probably participated in the ABA-mediated way of adversity response. To further study these two proteins characteristics, subcellular localization analysis was performed. The *ZmPHD14-GFP* and *ZmPHD19-GFP* fusion constructs and the GFP control driven by CaMV 35S promoter were introduced into tobacco epidermal cells. As shown in Figure 9B, the GFP signal was consistently observed throughout the whole cell, whereas ZmPHD14-GFP and ZmPHD19-GFP fusion proteins were restricted to the nucleus as confirmed by DAPI staining. In addition, in the onion epidermal cells, the GFP signals of ZmPHD14-GFP and ZmPHD19-GFP were only found in the nucleus, while the control GFP signals were widely distributed (Figure S2). These results both indicated that ZmPHD14 and ZmPHD19 were nuclear proteins, which was similar to GmPHDs [21].

## 3. Discussion

PHD-finger proteins have been identified in different plant species as involved in various biological processes, including the regulation of chromatin structure and transcription, response to adversity conditions, phosphate deficiency, DNA replication and so on. Although the number of PHD-finger genes that have been identified is increasing in different species, most of putative PHD family members remain unrecognized and uncharacterized and few comprehensive analyses of the PHD family in evolution had been conducted. In the present study, we performed a comprehensive genome-wide analysis of PHD family in maize. A total of 67 maize non-redundant *PHD* genes were identified. Previous study demonstrated that the maize genome size is approximately 18 times larger than *Arabidopsis*, while the maize gene number is about 1.3 times higher than *Arabidopsis* [23]. In *Arabidopsis*, a total of 45 *PHD* genes were identified from its genome [35]. However, the number of maize *PHD* genes was about 1.39 times higher than that in *Arabidopsis*, which was agreement with previous research (1.3) [23] and partially accounted for the support of PHD conservation in these two species during the evolutionary process.

Gene structure analysis indicated that most maize *PHD* genes within the same group shared similar organization, including exon/intron distribution and motif components, while divergence also existed among different groups, which might relate to functional diversity of maize PHD members (Figure 1 and Figure 3). In addition, of the 13 *ZmPHD* paralogous pairs, most shared conserved gene structure as well as the motif composition, while several pairs exhibited certain degrees of divergence. For example, the *ZmPHD65* contained five introns, whereas its counterpart *ZmPHD66* only possessed four introns. This divergence was also observed between *ZmPHD14* and *ZmPHD63* (Figure 1), which might be attributed to the single intron loss or gain during the evolution process in gene structure. In summary, the same group maize *PHD* genes were conserved in evolution, and also accompanying the variance of gene organization in some degree, suggesting that some maize PHD members were functionally diversified through differential expansion. In general, the analysis of maize *PHD* genes was kept in line with the phylogenetic analysis, and the consistency of gene structure and phylogenetic analysis also demonstrated the reliability of the phylogenetic analysis as well as the conserved evolutionary relationships among ZmPHD proteins.

Gene duplications are one of the primary driving forces in the evolution of genomes and genetic systems, including segmental duplication, tandem duplication, transposition events and whole-genome duplication [36], which had been demonstrated to play crucial roles in gene family expansion in many species, such as the HD-Zip and HSF family in maize, the Cyclophilin family in soybean and the WRKY family in cotton [22,37,38,39]. It was observed that many *PHD* genes in maize had one or more paralog genes, which implied that the expansion of the maize PHD family might be due to the gene duplications. It was believed that segmental duplication often occurred in a gene family that evolved slowly, like the *MYB* gene family [29], whereas, tandem duplication in local genomic clusters with low retention is common in the large and rapidly evolving gene family, like the Nucleotide binding site-leucine-rich repeats (NBS-LRR) disease resistance family [29]. Our analysis revealed that the number of maize *PHD* genes involved in segments duplication (12 pairs) was much larger than those arranged in chromosomal tandem duplication events (1 pair) (Table 2). Therefore, we hypothesize that the maize *PHD* gene family is a slowly evolving gene family, and that the segment duplications have played a key role in the expansion of the maize *PHD* gene family, which was also observed in some other maize gene families, such as the mitogen-activated protein kinase (MAPK) and CCCH-type zinc fingers [24,40]. It is hard to achieve the amplification of transcriptional regulating gene family through single-gene duplication events, which indicate the significance of genome duplications in the process of expanding the regulatory gene repertoire [41]. During the last 150 million years, more than 90% increases in regulatory genes were attributed to genome duplication in the *Arabidopsis* lineage [42]. Studies for the maize genome indicated that its genome had undergone at least two rounds of genome duplication, one ancient event was before rice diverged from the common ancestor of maize and sorghum at about 50 Mya, the other one was a recent one at approximately 11.9 Mya [43]. The duplication dates of maize *PHD* duplicated gene pairs were ranged from 7.38 to 74.53 million years (Mya) (Table 2), and the dates of four gene pairs were close to the first whole genome duplication Event (WGD) at 50 Mya, including *ZmPHD15/55*, *ZmPHD40/55*, *ZmPHD48/39* and *ZmPHD48/51*. Similarly, the dates of *ZmPHD14/63*, *ZmPHD22/30*, *ZmPHD37/56*, and *ZmPHD40/50* were close to the recent WGD date, which indicated that the large-scale genome duplication events also had played crucial roles in the expansion of maize PHD family. This phenomenon was also reported in *Gossypium raimondii*, where the average duplication date of *GrTCP* genes was very close to the recent whole-genome duplication date of *G.* *raimondii*, suggesting that large-scale genome duplication events might also contribute to the expansion of the GrTCP family [44]. Generally, the value of *K*a/*K*s > 1 indicated positive selection that accelerated the evolution; the *K*a/*K*s ratio = 1 signified neutral selection; while *K*a/*K*s ratio < 1 stood for negative selection or purifying selection [45]. By estimating the value of *K*a/*K*s for 13 duplicated gene pairs in maize, we found that the duplicated *ZmPHD* genes were under strong purifying selection during the history of evolution with an average *K*a/*K*s ratio of 0.286 (Table 2).

Previous study indicated that rice diverged from the common ancestor of maize, sorghum and rice at approximately 50 million years ago, which was followed by the divergence of maize and sorghum at 11.9 million years ago [31]. Our studies showed that 71 orthologous gene pairs were identified between maize and sorghum, whereas, only 54 orthologous gene pairs were found between maize and rice (Figure 5, Tables S2 and S3), which indicated that *ZmPHDs* were more closely allied with *SbPHDs* than *OsPHDs*; this phenomenon was also observed in maize peroxidase family [46]. Some *PHD* genes in maize had more than one counterpart in sorghum or rice, suggesting that *PHD* genes may undergo differential amplification in these three species. Similarly, 18 sorghum and nine rice PHD members shared one-more orthologous relationships with maize *PHD* genes, such as *Sb10g005910.1* to *ZmPHD56*, *-67* and *LOC_Os01g06540.1* to *ZmPHD39*, *-48* (Figure 5, Tables S2 and S3). These results indicated the conserved evolutionary relationship among maize, sorghum and rice, as well as the expansion of PHD family resulted from the whole-genome duplicated events.

According to the microarray expression profile analysis, these newly identified *PHD* genes exhibited distinct temporal and spatial expression patterns in different tissues or organs. This result indicated that these genes might be expressed under specific environments or at specific developmental stages. In addition, some duplicated *ZmPHD* gene pairs also showed diverse expression profiles between each other, which revealed that functional diversification of the surviving duplicated genes was a main feature of the long-term evolution [47]. Many PHD proteins had been detected in plenty of species to be involved in numerous physiological and biochemical processes, and their main functions was the focus on the regulation of chromatin structure and transcription, whereas, PHD members in response to aboitic stress in plant was rarely reported. Up to now, we found six soybean PHD proteins that conferred *Arabidopsis* salt tolerance through ABA-mediated signal transduction pathway [21]. Protein structure comparison and phylogenetic analysis revealed that these GmPHDs share close relationships with maize group IX PHD members (Figure S1), indicating the potential function of *ZmPHDs* in response to aboitic stress in maize, which was supported by qPR-PCR results that 15 maize *PHD* genes exhibited different degree response to ABA, NaCl and PEG treatments. Previous study indicated three outcomes of duplicated genes: neofunctionalization, subfunctionalization and pseudogenization [45]. Studies for 10 pairs of paralogous genes showed that tandem duplicated genes, *ZmPHD65* and *-66*, were not expressed during the whole treatment stage under three stress treatments. In addition, microarray expression profile analysis also revealed that both of them had no expressing among all tissues, which indicated that *ZmPHD65*, *-66* might be expressed under special conditions or at specific developmental stages or had undergone pseudogenization; this phenomenon was also detected in the study of soybean cyclophilin family. Of segmental duplicated genes, most exhibited similar expression profiles under certain stress treatment, such as *ZmPHD14/63*, *ZmPHD40/50* following PEG treatment, *ZmPHD39/51* following NaCl treatment (Figure 8), which indicated that the duplicated genes might have redundant functions in response to specific stress. However, the divergence was also existent between duplicated genes, suggesting neofunctionalization or subfunctionalization of duplicated genes, which was the major feature of most duplicated genes [47].

ABA plays a key role in plant adaptation to adverse environmental conditions. Lots of functional genes have been reported to be involved in plant abiotic stress response through ABA-mediated signal pathways, such as *OsMYB2*, *ZmMPK7* [48,49]. In the present study, six genes (*ZmPHD7*, *-14*, *-15*, *-19*, *-30*, *-39*) were strongly induced under ABA treatment (>3-fold) (Figure 8), indicating they might participate in the ABA signal pathway, which was supported by the promoter *cis*-element analysis (Figure 6). In addition, *ZmPHD14* and *ZmPHD19* also were highly induced or repressed under NaCl and PEG stress treatments. Previous studies showed that most PHD proteins were located in the nucleus. Subcellular localization analysis demonstrated that ZmPHD14 and ZmPHD19 were also nuclear proteins (Figure 9 and Figure S2), which was in accord with GmPHDs [21]. According to these results, we speculate that ZmPHD14 and ZmPHD19 and GmPHDs may share similar functions in response to abiotic stress conditions, and these remain to be further confirmed experimentally.

## 4. Experimental Section

### 4.1. Collection and Classification of PHD-Finger Transcription Factors

Identification and annotation of *PHD*-finger genes were performed in maize. First of all, we downloaded the Hidden Markov Model (HMM) profile of PHD-finger proteins from Pfam (Available online: http://pfam.sanger.ac.uk/search). The consensus protein sequences of the PHD-finger Hidden Markov Model profile were employed as a query to search against maize genome database with BLASTP program (Available online: ftp://ftp.ncbi.nlm.nih.gov/blast/executables/blast+/LATEST) [50]. Based on the results of further analysis for sequence alignment, chromosomal location and sequence identification numbers, all redundant protein sequences were discarded. To confirm the putative *PHD* genes in maize genome, the amino acid sequences of all the proteins were searched for the presence of PHD domains by Pfam and SMART (Available online: http://smart.embl-heidelberg.de/).

### 4.2. Phylogenetic and Gene Structure Analysis

Multiple sequence alignments for the 67 maize PHD-finger protein sequences were conducted using ClustalX version 1.83 with default settings [51]. Based on alignments, then an unrooted phylogenetic tree was generated by MEGA 4.0 (Available online: http://www.megasoftware.net/) using neighbor-joining (NJ) method with bootstrap analysis (1000 replicates) [52,53]. Another unrooted tree was constructed using the same method with the alignment of 17 maize PHDs and six soybean PHD protein sequences. Multiple sequence alignments were performed with BOXSHADE (Available online: http://www.ch. embnet.org/software/BOX_form.html) to highlight the conserved or similar amino acids residue location. The exon-intron organization analysis of the *PHD* genes was performed with Gene Structure Display Server (Available online: http://gsds.cbi.pku.edu.cn/) by comparing the CDS sequences with their corresponding DNA sequences. To identify the conserved motifs, the maize PHD proteins sequences were submitted to online Multiple Expectation maximization for Motif Elicitation (MEME) program (Available online: http://meme-suite.org/tools/meme) with the following parameters: any number of repetitions, the optimum width from 15 to 200 residues and maximum number of motifs 20. In addition, the indentified motifs were annotated with the Pfam and SMART programs [44].

### 4.3. The Analysis of Chromosome Location and Gene Duplication

Maize *PHD* genes were placed on 10 maize chromosomes according to their positions given in the TIGR maize database (Available online: http://maize.jcvi.org/repeat_db.shtml). The distribution of *ZmPHD* genes on the maize chromosomes was drawn by MapInspect (Available online: http://mapinspect.software.informer.com/) and modified manually with annotation. The tandem duplicated gene pairs and segmental duplicated gene pairs were identified according to previous studies [44,54]. The software MEGA 4.0 and DNAMAN 5.22 (Available online: http://dnaman.software.informer.com/) were used to analyses the homology of duplicated *ZmPHD* genes [55]. To further analyze gene duplication events, the nonsynonymous substitution rate (*K*a) and synonymous substitution rate (*K*s) were calculated by DnaSP 5.0 (Available online: http://www.ub.edu/dnasp) [56]. The ratios of *K*a/*K*s between each paralogs were estimated to detect the selection pressure in evolution. The *K*s value was used to count the dates of every duplicated events occurred in maize, based on a rate of λ substitutions per synonymous site per year. The duplicated time (T) = *K*s/2λ × 10^−6^ Mya (λ = 6.5 × 10^−9^ for grasses) [30,57].

### 4.4. Interspecies Microsynteny Analysis

To detect the syntenic regions among maize, sorghum and rice, the MCScanX (Available online: http://chibba.pgml.uga.edu/mcscan2/) was used [58]. First of all, the whole-genome protein sequences of maize, sorghum and rice were searched against themselves using BLASTPwith an *E*-value cutoff 1 × 10^−10^ and identity >75%. Subsequently, the MCScanX and associated downstream tools using default parameters were used for detecting the collinear blocks according to the previous study [58]. Finally, we identified the duplicated *PHDs* in these syntenic regions using in-house perl script, and the relationships of PHD orthologous genes among the three species were plotted using Circos software (Available online: http://circos.ca/) [59].

### 4.5. Cis-Elements in the Promoter Regions of Stress-Responsive PHD Class IX Genes

To predict *cis*-acting regulatory DNA elements (*cis*-elements) in promoter regions of maize *PHD* group IX genes, the PLACE website (Available online: http://www.dna.affrc.go.jp/PLACE/signalscan.html) was adopted to identify putative *cis*-elements in the 2000 bp genomic DNA sequences upstream of the initiation codon (ATG) [60].

### 4.6. Microarray-Based Expression Analysis of Maize PHD Genes

To analyze the spatial and temporal expression patterns of *PHD* genes during maize development, transcriptome data of the genome-wide genes expression atlas of maize inbred line B73 made by the NimbleGen microarray technology was downloaded from Plexdb (ZM37) (Available online: http://www.plexdb.org/modules/PD_browse/experiment_browser.php?experiment=ZM37). We got the transcriptome data from the article of Maize Gene Atlas Developed by RNA Sequencing and Comparative Evaluation of Transcriptomes Based on RNA Sequencing and Microarrays [61], the average expression value of a gene had to be greater than 0 FPKM (Fragments Per Kilobase Exon model per Million mapped fragments) in at least one of the 18 tissues, the RNA-Seq data were processed by adding a number 1. Then the microarray data of *PHD* genes were imported into R and Bioconductor (Available online: http://www.bioconductor.org/) for expression analysis.

### 4.7. Plant Materials and Stress Treatment

The maize inbred line B73 was used to examine the expression patterns of *PHD*-finger genes in all experiments. Plants grew in a greenhouse at 28 °C with a photoperiod of 14-h light/10-h dark and relative humidity 60% ± 5%. The soil was mixed containing soil/vermiculite/perlite as the volume proportion of 4:1:1. Distilled water was used to water the plant before treatment. Three-weeks-old B73 seedlings were treated under salt, PEG6000 and ABA stress. For ABA treatment, three-week-old seeding leaves were sprayed with 100 μM ABA solution, followed by sampling at 0, 1, 3, and 6 h [62,63]. Seedlings at the same stage were watering with 20% PEG6000 and 150 mM sodium chloride (NaCl) solution, followed by sampling at 0, 1, 3 and 6 h, which simulated the drought and salt stress, respectively. Seedlings without treatment were used as the control. For all the samples, three biological replicates were conducted.

### 4.8. RNA Isolation and qRT-PCR Analysis

Total RNA of all the samples was isolated using the Trizol reagent (Invitrogen, Foster city, CA, USA) according to the manufacturer’s instructions and the RNAs were treated with the DNase (Invitrogen). The first-strand cDNA was then synthesized using M-MLV reverse transcriptase (Invitrogen). The gene-specific primers were designed to amplify 80–150 bp PCR products by Primer Express 3.0 software (Applied Biosystems, Foster City, CA, USA), and their specificity were examined through Primer Blast on the NCBI (Table S1). Real-time PCR was performed on an ABI 7300 Real-Time system (Applied Biosystems). The total reaction volume was 20 μL contained 10 μL of SYBR Green Master Mix reagent (Applied Biosystems), 1.5 μL of cDNA sample, 1 μL of each gene-pecific primer, and 6.5 μL. The PCR condition was set as follows: 95 °C for 10 min, 40 cycles of 95 °C for 15 s and 60 °C for 1 min. Each sample was performed four technical replicates. The expression level of maize *GAPDH* gene was served as an internal control. The relative expression level was calculated as 2^−∆∆*C*t^ (∆*C*t = *C*t_Target_ − *C*t_GAPDH_, ∆∆*C*t = ∆*C*t_treatment_ − ∆*C*t_0 h_). The relative expression level (2^−∆∆*C*t0 h^) in the control plants without treatment was normalized to 1 as described previously [64]. Statistical analyses were performed using SDS software 1.3.1 (Applied Biosystems), and SPSS 19.0 (Avaliable online: http://www.spss.com.cn/).

### 4.9. Determination of Subcellular Localization of ZmPHD14 and ZmPHD19

Full-length cDNA of *ZmPHD14* and *ZmPHD19* were PCR-amplified and sequenced (Figure 9A), the primers were shown in Table S5. These two cDNA sequences were cloned between *Xba*I and *Bam*HI sites of the pCAMBIA1305-GFP vector (pCAMBIA1305-GFP is modified from pCAMBIA1305 vector). The resulting 35S:ZmPHD14-GFP, 35S:ZmPHD19-GFP and GFP control vector were transiently expressed in *Nicotiana benthamiana* leaves via *Agrobacterium*-mediated infiltration as described previously [65].Two days later, the fluorescence of the infected leaf tissue was observed under a Zeiss LSM700 (Zeiss, Jena, Germany) confocal microscope, the DNA dye 4,6-diamidino-2-phenylindole (DAPI) was used to visualize the nucleus. For the subcellular localization in onion, the Agrobacterium-mediated method was used. The onion cells were soaked in prepared Agrobacterium solution (OD_600_ = 1) for about 35 min and then placed on Murashige and Skoog (MS) medium. After 24 h of incubation at 25 °C in the dark, GFP fluorescence was observed with a fluorescence microscope (LEICA, Wetzlar, Germany).

## 5. Conclusions

We identified 67 non-redundant PHD members in the maize genome. Further analyses for phylogenetic relationships, gene structure and duplication, indicates that the conservation of the maize PHD family in evolution was accompanied with divergence to a certain degree. In addition, the expansion of the PHD family occurred, resulting from both large-scale genome duplication and small-scale duplicated events, such as segmental duplication and tandem duplication. Expression analysis demonstrated that group IX genes, especially, *ZmPHD14* and *ZmPHD19* are likely involved in the abiotic stress response. From an applied perspective, the identification of *ZmPHDs* will serve a very useful reference for more detailed functional analysis, and be useful in selecting appropriate candidate genes for further study of *PHD* genes in maize.

## Supplementary Materials

Supplementary materials can be found at http://www.mdpi.com/1422-0067/16/10/23517/s1.
