# Supplementary material for: Systematic Analysis of the Maize PHD-Finger Gene Family Reveals a Subfamily Involved in Abiotic Stress Response
Source: Int J Mol Sci. 2015 Sep 30;16(10):23517–44. doi: 10.3390/ijms161023517 (PMC4632711; doi:10.3390/ijms161023517)
Supplement: Supplementary file 1 [file ijms-16-23517-s001.zip › ijms-94615-Supplementary Information/ijms-94615-Tables S1, S3-S5 and Figures S1, S2.pdf]

Supplementary Information

Table S1. The sequence of 20 identified motifs.

Motif	Width	Conserved Amino Acid Sequences
1	72	KENLCLYGYPNETWEVNLPAEEVPPELPEPALGINFARDGMQEKDWLSLVAVHSDAWLLSVAFYFGARFGFD
2	41	FWICCDICEKWFHGKCVKITPAKAEHIKQYKCPSCSNKRAR
3	39	HYNPRTVEDVFRDFRGRRAGMIKALTTDVEKFYQQCDPE
4	43	CYICWDYGKMICCDRCDWWYHTYCMGPPHEQVPWGWWYCPCCC
5	49	KRLFTMINNLPTIFEVVTGTAKKQNKEKNPNSNHSKNKSKSKMQTHEEP
6	28	EEDLEEEDDEEHQNTLCGTCGENYGADE
7	200	ISIFVKDAICNDQCLGRCLYIHGVPGTGKTMSVLAVMRRLRSEFDSGTLRPYCFIEINGLKLASPENIYKVVYEQLSGHRVGWKKALHYLTEHFSGGTKIGKQANQPIILLIDELDLLMTRNQSVLYNILDWPTKPNSNLVVIGIANTMDLPEKLLPRISSRMGIQRLCFGPYNYRQLQEIITSRLKGIDAFEEQAIEFA
8	100	CNMKLAVALTVLDECFIPMVDPRTGIDMIHNVVYNCGSNFARLDFRGFYVVILEKGDEIIAVASIRIHGTKLAEMPFIGTRFMYRRQGMCRRLMNGIEKM
9	200	WAARIESLWREPDGTFWAKVRWYIIPEETAAGRQLHNLRRELYRTNDLADIEMETILRHCSIMCPKDFRDANDGGDDVFYCEYEYDIHWHNFKRLADIGDEPETKEDPSDEPYNAGDDYNSDTDEDSEYDEEEEPTSSFSVRGNQSHELAANSRKGRIYGLQKIGIQKIPEHVRCHQKTELEKAKATLLLATLPKSLPCR
10	200	CKPKREGSGKKEDFYWPQDFVLGDVVWARSGKKSPMWPALVIDPLQHAPEVVLNSCVPGALCVMFFGYSANGHGRDYGWVKQGMIFPFVDYLDRFQGQPLYKLRPTKFRAAIEEAFLAERGFFDLEMDGVCSPRKFVDKQSDPNGFQEEAASNNEQECQSESQVVGKSALCCDSCGNRLPSKVSKKRKQGGEQMLCMHCE
11	20	LWCQCYRCRTWYHPICYHQW
12	55	CCHKGGGAMPTTVDGKWVHVFCAWWQPEVYFPDMETMEPIMGIMNIQHNYFKCTC
13	55	CKICGGCEDEDKKFLICGHGLCSYKYYHILCLKEMQIASEVQRGLKCWYCPSCLC
14	152	CRRAAEFADYRVKQSRQSAQNTVSANKGDGVVSMGDIEAAIQEVFQAPHIQVMKNCPKFGKVILVAMVHELYKSGLGEIMFDKLATTVFSWCRANRELLPGYDTLLKICCKLGESKIILCEEGTKHKLQKVQLNYPSDDVTFALKESPDLPW
15	28	SWNTSYEQRMFMSPTTISREVGPPGYGP
16	200	ALRFNGWHPTEHGNLVDDGGTGEEDPVGAVHSEENSGALVQLEDGTVAPLEANKTMCHDTLFDILISEKFAMLCDLLAATFHGSKPDDVIGLQIIDAKMRNGDYAQNHALFDHDIKQIWKKFEHVGQEMAGLASSLLVISQASNQKQASGISEIDVAEHKIEETSLVGVTRKALREFTPQCDSGYSTIPKRTGRSGSDGI
17	89	DNSWTWWTGGNISKRTLQRGALLHSTIRKAARQGGKRRIAGLSYHEGSQFPRRSRQFFWRACVGISQTSSQLALQVRYLDWHIRWKEFI
18	200	FKPQAYMNLYNHGNIAASAAANIAIITSDEGKVSTSQLTAKHKKKMAADNALQLKAFSSAVTQFVWPSTEKKLMEVPRDRCGWCLACRSSAIGNKKACFLNMTTSNAVKCSARILSVMRVIKNSDSHFPSIVAYLANMEESLRGLLVGSLQDMQQKERWRQQLQEASNCRTVIPLLLELESNIRGVAFSASWFKPIDDWP

Table S1. *Cont.*

Motif	Width	Conserved Amino Acid Sequences
19	200	NSCLGDQTIDGNHDMSPFKNDDNVYISSSTRSLEKNLKSPSMKAVTNNADMI<br>PKVKIKGSKVSSLHYKDGEENTPKNDTGKATKLVIHLGSRHKTRSGSPKSELS<br>NYQREQDLGSIHGGKIDVTSQLKGSRNEVKERSVMKLVRETGVQQRNSLLGD<br>LGTSKKHATGKRSNALISGMENGNETGTRNRPFAQKQSHSSQV
20	112	SNSSDFTKEEPSEPCPPVQAVELPPSSGDIHVPEDSISYLFSVYNFLRSFSVQLFL<br>SPFGLDDFVAAINCTVQNNLLDWVHVSLLRALRRHLESKSAEGSQSASNCLK<br>YLDW

Table S3. The information of orthologous genes among maize, rice and sorghum in Figure 5.

Gene Name	Sequence ID	Orthologous Sorghum	Gene Name	Sequence ID	Orthologous Rice
ZmPHD1	GRMZM5G862565	Sb01g028420.1	*ZmPHD1*	GRMZM5G862565	LOC_Os10g42196.1
ZmPHD3	GRMZM5G813111	Sb01g008160.1	*ZmPHD3*	GRMZM5G813111	LOC_Os03g53700.1
ZmPHD4	AC225147.4	Sb01g003420.1	*ZmPHD4*	AC225147.4	LOC_Os03g60400.1
		Sb01g003410.1			LOC_Os03g60390.1
ZmPHD5	GRMZM2G403562	Sb05g002930.1	*ZmPHD5*	GRMZM2G403562	LOC_Os11g05130.1
		Sb08g002920.1	*ZmPHD7*	GRMZM2G047316	LOC_Os07g12910.1
ZmPHD7	GRMZM2G047316	Sb02g006980.1	*ZmPHD8*	GRMZM2G409224	LOC_Os09g38440.1
ZmPHD8	GRMZM2G409224	Sb02g032470.1	*ZmPHD10*	GRMZM2G158194	LOC_Os01g08820.1
ZmPHD10	GRMZM2G158194	Sb03g003580.1	*ZmPHD11*	GRMZM2G085266	LOC_Os01g11952.1
ZmPHD11	GRMZM2G085266	Sb03g001640.1	*ZmPHD15*	GRMZM2G080917	LOC_Os01g66420.1
ZmPHD12	GRMZM2G069886	Sb08g019950.1			LOC_Os05g34640.1
ZmPHD13	GRMZM2G081350	Sb08g016700.1	*ZmPHD18*	GRMZM2G068331	LOC_Os11g12650.1
ZmPHD14	GRMZM2G153087	Sb05g007010.1	*ZmPHD19*	GRMZM2G008259	LOC_Os11g14010.1
ZmPHD15	GRMZM2G080917	Sb03g042180.1	*ZmPHD21*	GRMZM5G871463	LOC_Os08g32620.1
		Sb09g020610.1			LOC_Os09g21770.1
ZmPHD16	GRMZM2G025703	Sb03g034510.1	*ZmPHD22*	GRMZM2G107807	LOC_Os02g35600.1
		Sb09g025810.1			LOC_Os04g36730.1
ZmPHD17	GRMZM2G314546	Sb03g034510.1	*ZmPHD23*	GRMZM2G067019	LOC_Os02g39800.1
		Sb09g025810.1	*ZmPHD24*	GRMZM2G385338	LOC_Os02g09920.1
ZmPHD18	GRMZM2G068331	Sb05g007460.1	*ZmPHD27*	GRMZM2G087482	LOC_Os03g53630.1
ZmPHD19	GRMZM2G008259	Sb05g007010.1	*ZmPHD29*	GRMZM2G466270	LOC_Os06g51490.1
		Sb08g006530.1	*ZmPHD30*	GRMZM2G063864	LOC_Os02g35600.1
ZmPHD20	GRMZM2G059266	Sb07g000565.1			LOC_Os04g36730.1
ZmPHD21	GRMZM5G871463	Sb02g023940.1	*ZmPHD31*	GRMZM2G045544	LOC_Os02g48800.1
		Sb07g020610.1			LOC_Os06g20410.1
ZmPHD22	GRMZM2G107807	Sb04g023220.1	*ZmPHD33*	GRMZM2G039895	LOC_Os08g01420.1
		Sb06g017810.1	*ZmPHD37*	GRMZM2G168249	LOC_Os06g08790.1

Table S3. *Cont.*

Gene Name	Sequence ID	Orthologous Sorghum
ZmPHD22	GRMZM2G107807	Sb04g023220.1
		Sb06g017810.1
ZmPHD23	GRMZM2G067019	Sb04g025725.1
ZmPHD24	GRMZM2G385338	Sb04g006240.1
ZmPHD27	GRMZM2G087482	Sb01g008195.1
ZmPHD28	GRMZM2G466292	Sb10g031260.1
ZmPHD29	GRMZM2G466270	Sb10g031265.1
ZmPHD30	GRMZM2G063864	Sb04g023220.1
		Sb06g017810.1
ZmPHD31	GRMZM2G045544	Sb10g011813.1
		Sb04g029710.1
ZmPHD32	GRMZM2G368206	Sb04g034380.1
ZmPHD33	GRMZM2G039895	Sb07g000565.1
ZmPHD34	GRMZM2G134214	Sb10g000260.1
ZmPHD35	GRMZM2G103230	Sb10g001106.1
ZmPHD36	GRMZM2G473258	Sb10g026360.1
ZmPHD38	GRMZM2G330024	Sb10g006890.1
		Sb04g034380.1
ZmPHD39	GRMZM2G148810	Sb03g005320.1
		Sb09g004740.1
ZmPHD40	GRMZM2G016817	Sb09g020610.1
ZmPHD41	GRMZM2G412492	Sb02g004100.1
ZmPHD42	GRMZM2G097726	Sb02g004890.1
ZmPHD44	GRMZM2G091265	Sb02g023940.1
		Sb07g020610.1
ZmPHD45	GRMZM2G316191	Sb02g033850.1
ZmPHD46	GRMZM2G128176	Sb02g042000.1
ZmPHD47	GRMZM2G434715	Sb02g043960.1
		Sb01g038485.1
ZmPHD48	GRMZM5G893976	Sb03g005320.1
		Sb09g004740.1
ZmPHD49	GRMZM2G372928	Sb03g001640.1
ZmPHD50	GRMZM2G158918	Sb09g020610.1
ZmPHD52	GRMZM2G335720	Sb03g025310.1
ZmPHD53	GRMZM2G170412	Sb03g029850.1
ZmPHD54	GRMZM2G149587	Sb03g046950.1
ZmPHD56	GRMZM2G455243	Sb10g005910.1
ZmPHD58	GRMZM2G472428	Sb10g000310.1
ZmPHD59	GRMZM5G889372	Sb01g037770.1
ZmPHD60	GRMZM2G178072	Sb01g047350.1
ZmPHD61	GRMZM2G314661	Sb05g002930.1
ZmPHD63	GRMZM2G115424	Sb08g006530.1

Gene Name	Sequence ID	Orthologous Rice
ZmPHD33	GRMZM2G039895	LOC_Os08g01420.1
ZmPHD37	GRMZM2G168249	LOC_Os06g08790.1
ZmPHD38	GRMZM2G330024	LOC_Os02g52960.1
		LOC_Os06g10690.1
ZmPHD39	GRMZM2G148810	LOC_Os01g06540.1
		LOC_Os05g07040.1
ZmPHD41	GRMZM2G412492	LOC_Os07g07690.1
ZmPHD42	GRMZM2G097726	LOC_Os03g58530.1
		LOC_Os07g08880.1
ZmPHD44	GRMZM2G091265	LOC_Os08g32620.1
		LOC_Os09g21770.1
ZmPHD46	GRMZM2G128176	LOC_Os07g46690.1
ZmPHD47	GRMZM2G434715	LOC_Os07g49290.1
ZmPHD48	GRMZM5G893976	LOC_Os01g06540.1
		LOC_Os05g07040.1
ZmPHD49	GRMZM2G372928	LOC_Os01g11952.1
ZmPHD50	GRMZM2G158918	LOC_Os05g34640.1
ZmPHD52	GRMZM2G335720	LOC_Os01g36670.1
ZmPHD53	GRMZM2G170412	LOC_Os01g46700.1
ZmPHD54	GRMZM2G149587	LOC_Os01g73460.1
ZmPHD56	GRMZM2G455243	LOC_Os06g08790.1
ZmPHD57	GRMZM2G156129	LOC_Os06g01170.1
ZmPHD59	GRMZM5G889372	LOC_Os03g19020.1
		LOC_Os07g49030.1
ZmPHD60	GRMZM2G178072	LOC_Os03g04980.1
ZmPHD62	GRMZM2G365888	LOC_Os12g34330.1
ZmPHD65	GRMZM2G156088	LOC_Os04g36730.1
ZmPHD66	GRMZM2G050495	LOC_Os02g35600.1
ZmPHD67	GRMZM2G038050	LOC_Os04g59510.1

Table S3. *Cont.*

Gene Name	Sequence ID	Orthologous Sorghum	Gene Name	Sequence ID	Orthologous Rice
ZmPHD64	GRMZM2G404426	Sb07g006270.1			
ZmPHD65	GRMZM2G156088	Sb06g017810.1			
ZmPHD66	GRMZM2G050495	Sb04g023220.1			
ZmPHD67	GRMZM2G038050	Sb06g034065.1			

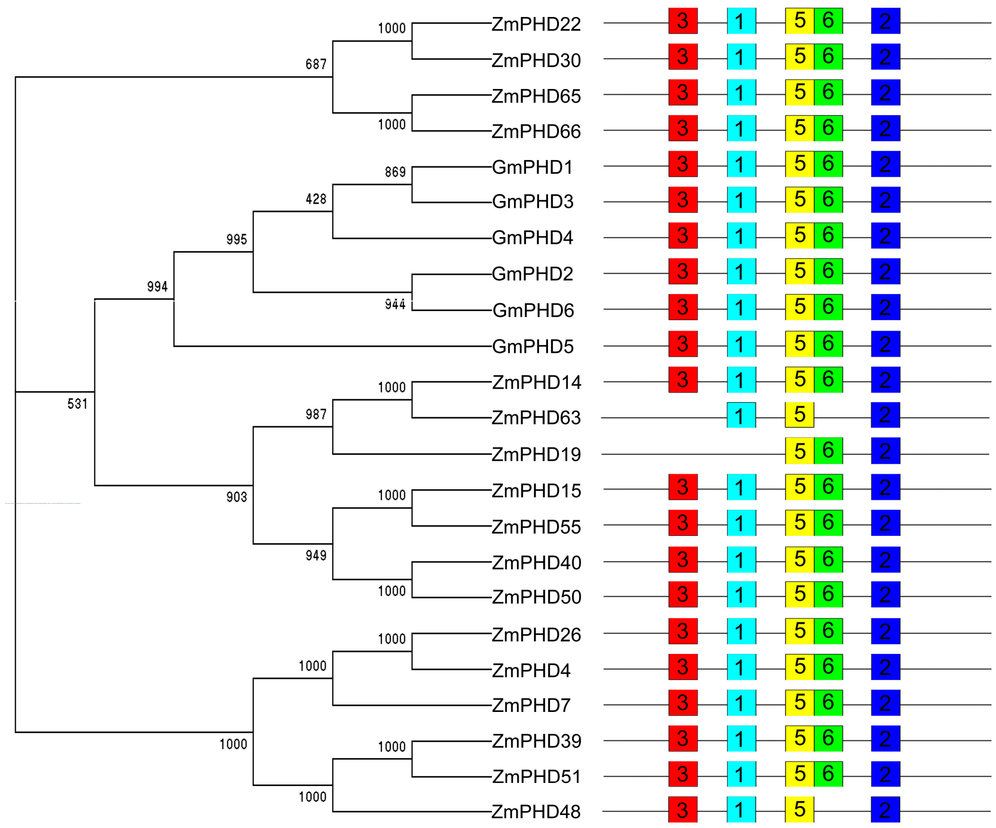

Figure S1. Phylogenetic relationship and motifs of 17 ZmPHD and 6 GmPHD proteins. The Phylogenetic tree is generated by MEGA software, and all the conserved motifs are identified by online MEME program. Motif 2 represents the PHD domain and other motifs are functions unknown.

Table S4. The number of *cis*-element in the promoters of group IX genes.

Gene Name	ABRE Number	DRE Number	MYBE Number
ZmPHD4	2	1	7
ZmPHD7	1	1	1
ZmPHD14	3	3	6
ZmPHD15	1	2	1
ZmPHD19	6	0	3
ZmPHD22	4	1	1
ZmPHD26	7	1	3

Table S4. *Cont.*

Gene Name	ABRE Number	DRE Number	MYBE Number
ZmPHD30	2	2	3
ZmPHD39	3	1	3
ZmPHD40	1	0	5
ZmPHD48	2	2	6
ZmPHD50	1	2	5
ZmPHD51	0	0	7
ZmPHD55	5	3	2
ZmPHD63	3	3	3
ZmPHD65	0	1	6
ZmPHD66	0	1	6

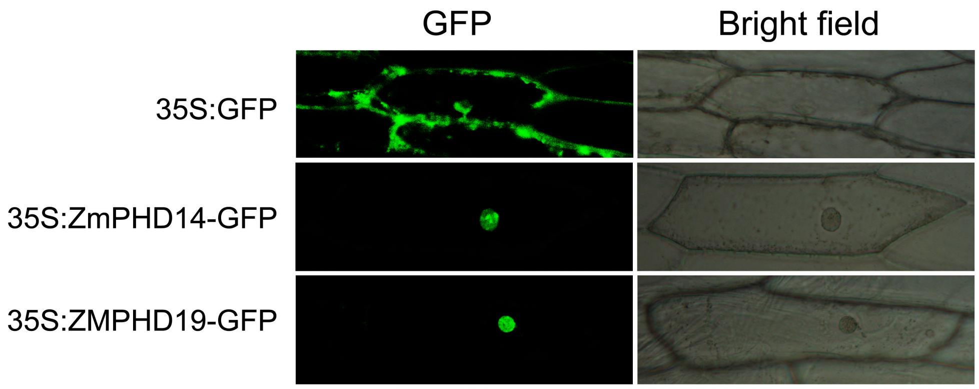

Figure S2. Subcellular localization of ZmPHD14-GFP and ZmPHD19-GFP in onion epidermal cells. The GFP fluorescence was visualized by fluorescence microscopy under bright-field and UV light. The 35S-GFP was as control. The green color are GFP protein signal.

Table S5. Maize *PHD* gene-specific primers used for qRT-PCR analysis.

Primer Name	Forward Primer (5′→3′) Sequence	Reverse Primer (5′→3′) Sequence
ZmPHD4	TCTGACTCGTGGCTCATCTCA	ATAAGCGCTTCCGGTCGTT
ZmPHD7	CACGGCAAATGTGTGAGGAT	TGCAATCAGGGCACTTGTAGTG
ZmPHD14	GATGGGATGGATGAAAAAGATTG	GCAACAGACATTAGCCAAGAATCA
ZmPHD15	GAGGAAGTGCCCCCAGAGA	ATGCCATCCCTAGCAAAGTTGA
ZmPHD19	GCAATAAAGCTGGCCCAAAA	GCATCTTCGAACCCCTTGAG
ZmPHD22	CAGAACCAGCACTGGGCATA	GAGACAACCAGTCTTTTTCAACCA
ZmPHD26	TCCCAACAGGACGGCTACA	TCGCGTGCCGGAGAGA
ZmPHD30	GGTCACTCTTCCAGCTGAGGAA	AAAGTTTATCCCCAGTGCTGGTT
ZmPHD39	GCGTTTGTTTGGCATGATGA	CCACCGGAGACGACTTCAA
ZmPHD40	CGGTTTGGGTTTGACAAAGAG	TTGTGGGCAAGTTATTTATCATGTTG
ZmPHD48	TCTCCGGTGCGAGACAACA	AGGCTTGGCTCTACCACCATT
ZmPHD50	TCCTGGTTACTAGCAGTTGCATTCTA	CGTCTGGCCTCTTTGTCAAAC
ZmPHD51	GCGTTTGTTCGGCATGATG	TCACACCACTGGAGACGACTTC

Table S5. *Cont.*

Primer Name	Forward Primer (5′→3′) Sequence	Reverse Primer (5′→3′) Sequence
ZmPHD55	TGGTGAGTGCGCTCGATCT	CTCCTCGGCTATCACTCCACAT
ZmPHD63	CTGTGCGGCGCATGTG	TCTCGCATAAGTCGCAACAGA
ZmPHD65	TGTGCGACCCAGAGTATAAGGA	TTCACTTCCCAGGTTTTGTTAGG
ZmPHD66	CGGCTCCACATGATGATCAA	CTCCCCACTCCCTATCACAACT
ZmGAPDH	CCCTTCATCACCACGGACTAC	AACCTTCTTGGCACCACCCT
